# Evaluating the Quality of Life of Beneficiaries After Providing Financial Aid by a Multi-specialty Tertiary Care Hospital

**DOI:** 10.7759/cureus.59320

**Published:** 2024-04-29

**Authors:** Rehana C Mukundan, Sanjeev Singh, Ajith kumar, T. V Sathianandan

**Affiliations:** 1 Patient Services, Amrita Institute of Medical Sciences and Research Center, Kochi, Kochi, IND; 2 Medicine, Amrita Institute of Medical Sciences and Research Center, Faridabad, Faridabad, IND; 3 Commerce and Management, Amrita School of Arts and Sciences, Kochi, Kochi, IND; 4 Biostatistics, Amrita Institute of Medical Science and Research Center, Kochi, Kochi, IND

**Keywords:** sleep, satisfaction, stress, health related expense, support programmes, health care services, multi-specialty, comparative analysis, financial aid, quality of life

## Abstract

Background

Financial aid programs offered by multi-specialty tertiary care hospitals play a crucial role in ensuring equitable access to healthcare. This study investigates the effect of financial aid on the quality of life (QoL) of beneficiaries, aiming to provide a comprehensive understanding of the multifaceted relationship between healthcare support and overall well-being.

Aim

The study's objectives included assessing changes in pre- and post-aid QoL, identifying influencing factors, understanding beneficiary experiences, and evaluating the effectiveness of financial aid programs.

Methods

The study adopted quantitative assessments through QoL questionnaires developed based on the WHO BREF questionnaire and insights obtained through interviews. A representative sample of beneficiaries was selected, informed consent was obtained, and an institutional ethical certificate was also obtained.

Results

The findings overwhelmingly support the alternative hypothesis. The alternative hypothesis was that after receiving financial support, recipients' quality of life would increase. Quantitative analysis revealed a statistically significant enhancement in the QoL of beneficiaries across physical, mental, and social well-being domains. The quality of life scores of patients before and after receiving the support was statistically tested using a paired t-test, and the quality of life score has improved significantly with a p-value of 4.156 × 10^-28^ (p value<0.001). The comparison of quality of life scores of the control group with the patient's group before getting the support was tested using an independent sample t-test and found to be non-significant (p=0.496), while a similar comparison between the control group and the patient's group after receiving the support was found to be statistically highly significant with a p-value of 8.721 × 10^-28^ (p-value<0.001).

Conclusions

This research demonstrates the substantial impact of financial aid on the QoL of beneficiaries in a multi-specialty tertiary care hospital setting. It underlines the importance of addressing economic barriers and providing patient-centered, holistic support. These insights have broader implications for healthcare policy and practice, promoting a more comprehensive approach to patient well-being.

## Introduction

In recent years, healthcare systems across the globe have recognized the important role of financial aid in improving the well-being and quality of life (QoL) of patients in need. Hospital financial assistance programmes are a critical component of the healthcare system, designed to alleviate financial burdens and enhance healthcare accessibility for low-income patients. This approach is not only a testament to the humanitarian ethos that underpins modern medicine but also an acknowledgment of the inextricable link between financial stability and overall health and well-being.

Multi-specialty tertiary care private hospitals are at the forefront of medical innovation and patient care. These institutions often cater to a diverse patient population, offering advanced treatments and comprehensive services across a spectrum of medical specialties. As a result, they frequently encounter patients who require complex, high-cost interventions that can place a significant financial burden on individuals and families. In response to this challenge, many such hospitals have implemented financial aid programmes to ensure that essential medical care remains accessible to all, regardless of their economic circumstances. This article aims to shed light on the crucial aspect of evaluating the QoL of beneficiaries after they have received financial aid from the Amrita Institute of Medical Sciences and Research Centre (AIMS), Cochin, India.

Quality of life, a multifaceted concept encapsulating physical, psychological, social, and environmental well-being, is inherently intertwined with health outcomes [1]. As per the World Health Organization (WHO), QoL is defined as "an individual's perception of their position in life in relation to their goals, expectations, standards, and concerns and in the context of the culture and value systems in which they live." This expansive notion is intricately shaped by an individual's physical and mental well-being, alongside their personal values, social connections, and interactions with significant elements of their environment. The holistic nature of QoL underscores the importance of considering diverse factors that contribute to an individual's overall sense of well-being, emphasizing the interconnectedness between health, personal values, and the broader socio-cultural context [2]. The correlation between financial security and enhanced QoL is firmly established, supported by research demonstrating that economic stability can yield positive effects on diverse facets of an individual's life. Growing evidence reveals that financial strains and worries play significant roles in mental health. This influence extends to areas such as physical and mental health, family dynamics, and overall life satisfaction, as evidenced by existing studies [3]. The acceptance of illnesses stands as a pivotal juncture in this narrative, wielding the power to influence not just the physical aspects of health but also the intricate interplay of emotions, relationships, and the fundamental perception of one's existence. This exploration delves into the multifaceted dimensions of how the acceptance of illnesses permeates the lives of patients, impacting their perspectives, emotional resilience, and the quality of their daily experiences [4]. The QoL of patients coming to this hospital can be affected by other factors like disease, treatment and surgeries, individual traits, and social circumstances. Against the backdrop of societal structures, individuals hailing from lower socio-economic backgrounds navigate a complex web of challenges where access to financial resources, educational opportunities, community engagement, and medical facilities is often constrained. This confluence of limitations emphasizes the critical importance of assessing the socio-economic landscape these individuals inhabit. This exploration ventures into the intricate relationships that intertwine with economic disparities, shedding light on the pressing need to scrutinize and understand the multifaceted dimensions of limited access. As we embark on this examination, we unravel the intricate ways in which socio-economic factors can shape and, in many cases, constrict the life experiences of those at the lower rungs of the economic ladder [5,6].

The objective of this study is to explore and analyse the impact of financial aid provided by a multi-specialty tertiary care hospital on the QoL of beneficiaries. Additionally, we seek to compare the QoL of patients who received financial assistance with those who did not. By delving into this matter, our aim is to augment the expanding body of knowledge concerning the wider repercussions of healthcare interventions that extend beyond immediate medical outcomes. Understanding how financial support influences the QoL of individuals not only serves as a testament to the hospital's commitment to patient welfare but also guides the development of more effective, patient-centred financial assistance programmes.

## Materials and methods

Study design

This was a prospective cross-sectional study conducted in AIMS, Kochi, that comprehensively assessed the impact of financial aid on the QoL of beneficiaries. The study incorporates quantitative data collection and analysis methods to provide a holistic understanding of the subject matter.

Ethical considerations

Ethical approval was obtained from the institutional ethical committee to ensure that the study adheres to ethical guidelines. Participants were provided with information about the study's purpose and procedures and were required to provide informed consent. Each and every study participant gave their informed consent. All data were anonymized to protect the privacy of the participants. Identifying information was removed or replaced with pseudonyms.

Sample selection

Since there is no specific sample size estimation for the regression model, as per the guidelines, the sample size was calculated considering 10 subjects per variable for a set of eight QoL variables. The study includes 80 subjects, with 46 males and 34 females. It includes 40 representative samples of beneficiaries who had received financial aid and 40 samples who had not received financial aid, as they are from the lower middle class/middle class. Five departments from AIMS Kochi, namely cardiology, gastro-intestinal surgery, neurosurgery, nephrology, and paediatric cardiology, were selected for the study because of the high costs of their treatments in the concerned departments. A simple random sampling method was used to select participants. To ensure diversity, the sample encompasses patients from various age groups, medical conditions, and socioeconomic backgrounds. To mitigate bias, participants with similar illness severity and age groups were carefully chosen for inclusion in the control group in the study. While the majority of participants hailed from socioeconomically disadvantaged backgrounds, individuals facing the most pronounced economic hardships were given precedence for financial assistance, distinguishing them from the test group, while the others formed the control group. The study encompassed individuals of all age groups from Kerala, spanning across its 14 districts, ensuring a comprehensive representation of the population for analysis.

Data collections and tools

The Institutional Scientific and Ethics Committee evaluated the research proposal, and clearances were obtained. The chiefs of several departments also provided their consent for the gathering of data. The attending physician diagnosed and recommended a procedure or surgery to a patient as soon as they arrived at the OP department. After that, a person who had information about the surgery went to the Financial Information Counter (FIC) and told the patient how much of the approximate expenses they had to pay. When patients expressed difficulty paying the expenses, the hospital sent them to the Patient Services Department, its charity wing. If the subjects belong to lower socio-economic status, they are eligible and receive financial aid; if not, they are referred to the FIC for proceedings. As per WHO guidelines, QoL assessment can be performed following a four-week period of treatment. Accordingly, 20 to 35-minute interviews were conducted with the 80 subjects. A questionnaire was used as the basis for an interview, and it was distributed four weeks before and after receiving financial aid. The questionnaire for QoL assessment was based on WHO guidelines, where each question had a scale with minimum and maximum scores. Each question assessed QoL factors with scores, and they were present condition, social life, health condition, concentration, capacity for work, physical appearance, sleep, need for money, satisfaction, and stress. It is employed to quantify QoL in terms of both its mental and physical components. It mainly focuses on the present condition, social life, need for money, experiences, and convictions of the respondents, as well as other aspects of their quality of life. A statistical analysis was then performed on all of these data.

Questionnaires

Before and after receiving financial aid, participants complete QoL assessment questionnaires to track changes in their physical, mental, and social well-being and to compare their results with those of the control group.

Medical records

Relevant medical data, including diagnoses and treatment details, are collected to account for the impact of the medical condition on QoL.

Both primary and secondary data were equally used in this study. The sample interview method was employed in order to obtain primary data. Through journals, websites, books, and other sources, secondary data were acquired.

Statistical analysis

IBM SPSS version 20.0 software was used to conduct the statistical analysis. Statistical analysis techniques, such as paired t-tests, are used to compare pre- and post-aid QoL scores, and independent sample t-tests are used to compare pre- and post-aid QoL scores with the QoL scores of the control group.

## Results

The socio-demographic characteristics of the patients are shown in Table 1.

**Table 1:** Socio-demographic characteristics of the subjects n=80

Demographic details	n (%)
Gender	
Male	46(57.5)
Female	34(57.5)
Age	
Above 50	32(40.0)
40–50	20(25.0)
25–40	16(20.0)
15–25	4(05.0)
0–15	8(10.0)
Place	
Northern Kerala	24(30.0)
Central Kerala	40(50.0)
Southern Kerala	16(20.0)

The study ensured comparability by recruiting patients with similar treatment procedures in both the test and control groups, with similar age and illness severity. While the socioeconomic status of participants was generally characterized as poor, those facing more significant economic challenges were prioritized for financial aid in the test group and the control group. This approach aimed to address the needs of individuals with the greatest economic difficulties while maintaining comparability across study groups, enhancing the fairness and relevance of the research findings.

Results on comparison of QoL scores before and after providing financial aid

The quality of life scores of patients before and after receiving the support were statistically tested using a paired t-test, and the quality of life score has improved significantly with a p-value of 4.156 × 10^-28^ (p<0.001). The comparison of quality of life scores of the control group with the patient's group before getting the support was tested using an independent sample t-test and found to be non-significant (p=0.496), while a similar comparison between the control group and the patient's group after receiving the support was found to be statistically highly significant with a p-value of 8.721 × 10^-28^ (p<0.001) (Tables 2-3).

**Table 2:** Quality of life scores: before versus after financial aid n=80

Paired samples statistics		Mean	N	Std. deviation	P-value
Pair 1	Pre-patient QLS	29.48	40	2.491	4.156E-045
	Post-patient QLS	43.13	40	1.343	

**Table 3:** Quality of life scores: financial aid recipients versus control group

Paired samples statistics		Mean	N	Std. deviation	P-value
Pair 1	Post-patient QLS	43.13	40	1.343	8.721E-048
	Control-patient QLS	29.08	40	2.314	

The overall experience of 40 beneficiaries, who had a QoL score of 109 before receiving financial aid, was found to have improved to 165.

**Figure 1:**
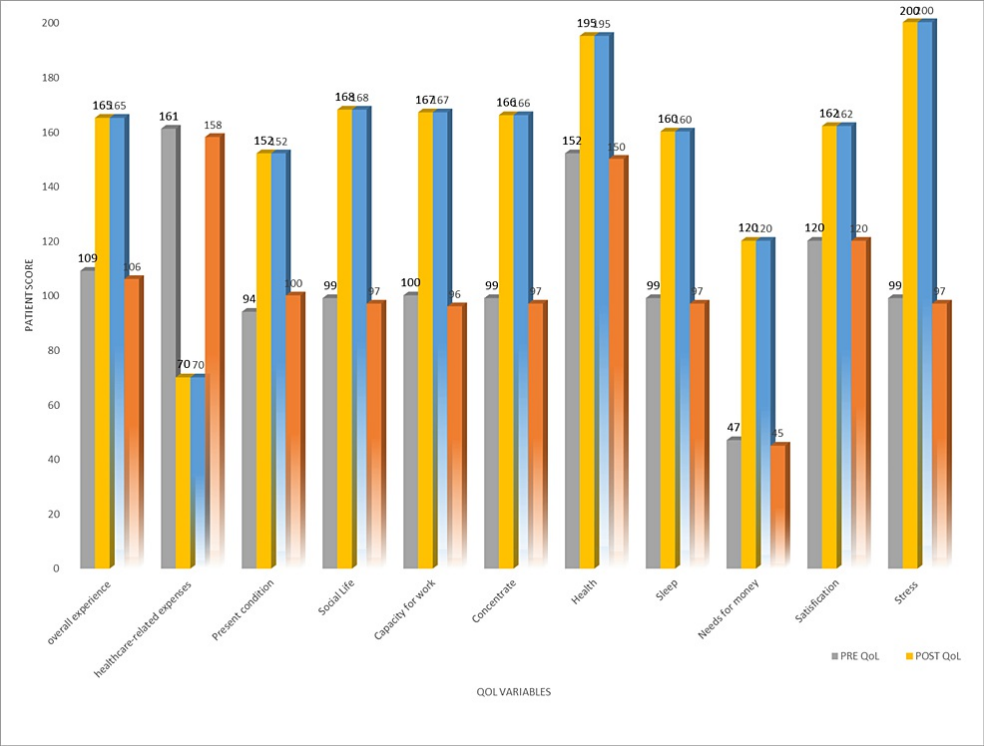
Quality of life evaluation before and after providing financial aid

Of the 40 beneficiaries prior to receiving financial aid, health-related expenses were 161, and they were found to have been reduced to 70. In the present condition of 40 beneficiaries before receiving financial aid, the QoL score was 94; it was later found to be improved to 152. Before receiving financial aid, the QoL score of 40 beneficiaries in social life was 99; it was later found to be improved to 168. Before receiving financial assistance, the 40 beneficiaries' QoL score in terms of their ability to work was 100; it was later found to have improved to 167. Before receiving financial aid, the QoL score of 40 beneficiaries for the concentration was 99; it was later found to be improved to 166. The QoL score of 40 beneficiaries in terms of their health was 152 before financial aid was provided, but it was found to have improved to 195. The QoL score of 40 beneficiaries in terms of their sleep was found to have improved to 160 from 99. The QoL score of 40 beneficiaries who needed enough money before providing financial aid was 47, and it was found to have improved to 120. Forty beneficiaries' QoL scores in terms of their satisfaction were 120 before financial aid was provided and were found to have improved to 162. The QoL score of 40 beneficiaries in terms of stress before providing financial aid was 99, and it was found to have improved to 200 (Figure 1).

**Figure 2:**
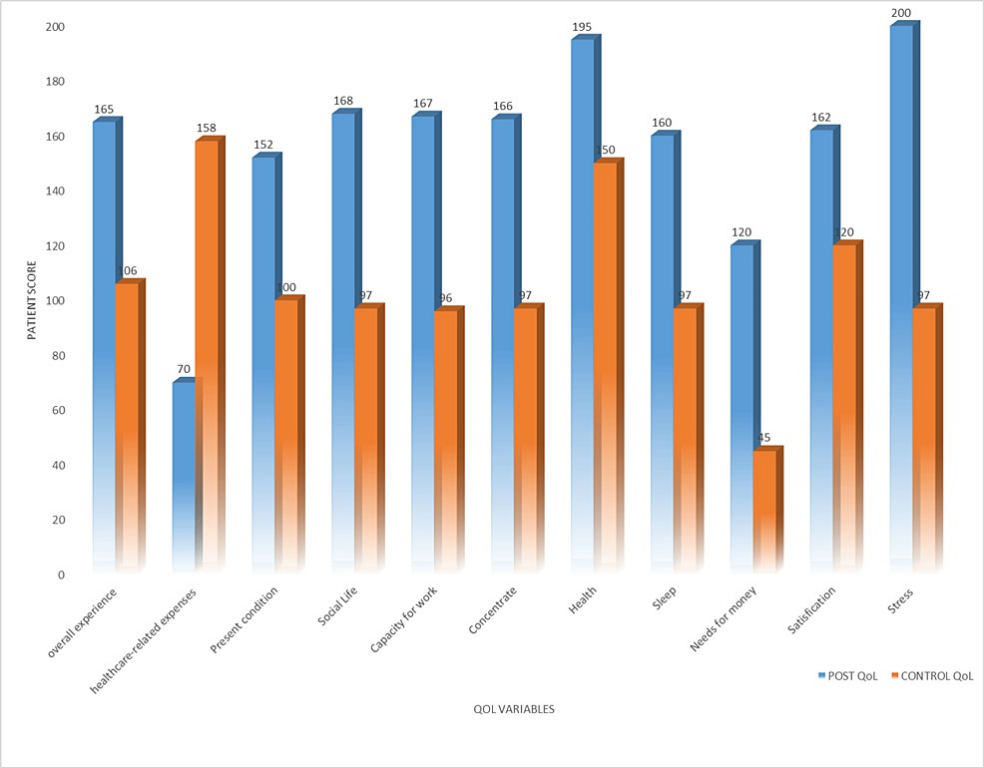
Comparative quality of life evaluation between beneficiaries and control group

The QoL score of 40 beneficiaries in the overall experience after providing financial aid was 165, and the QoL score of the other 40 people without getting financial aid (the control group) was 106. After receiving financial assistance, the QoL score of 40 recipients for health-related costs was 70, while the QoL score of the other 40 individuals (the control group) who did not get financial assistance was 158. After receiving financial aid, the QoL score of 40 recipients in the present condition was 152, while the QoL score of the other 40 individuals (the control group) who did not get financial aid was 100. After receiving financial aid, the QoL score of 40 recipients in their social lives was 168, whereas the QoL score of the other 40 individuals (the control group) who did not get financial aid was 97. After receiving financial assistance, the QoL score of 40 beneficiaries who had the capacity to work was 167, while the QoL score of the other 40 individuals (the control group) who were not receiving financial assistance was 96. After receiving financial assistance, the QoL score of 40 recipients in the concentration was 166, while the QoL score of the other 40 individuals (control group) who did not get financial assistance was 97. After receiving financial aid, the QoL score of 195 beneficiaries in health was 165, while the QoL score of the remaining 40 individuals (the control group) who did not get financial aid was 150. After financial aid was provided, the QoL score for sleep was 160, while the QoL score for the remaining 40 individuals (the control group) who did not get financial aid was 97. The QoL score of 40 beneficiaries in need of enough money after providing financial aid was 120, and the QoL score of the other 40 people without getting financial aid (the control group) was 45. Following the granting of financial aid, the QoL score in satisfaction of 40 beneficiaries was 162, while the QoL score of the other 40 individuals (the control group) who did not get financial aid was 120. After receiving financial aid, the QoL score of 40 beneficiaries who were under stress was 200, whereas the QoL score of the other 40 individuals (the control group) who did not get financial aid was 97 (Figure 2).

## Discussion

AIMS is a multispecialty tertiary charitable care hospital in Kochi, Kerala, established in 1998 by Sri Sri Mata Amritanandamayi Devi (popularly known as Amma), an adept spiritual and compassionate leader with the intention of providing exceptional and affordable medical care to all, regardless of colour, social class, religion, or financial situation. The institution aims to provide healthcare of the highest quality to all in a spirit of compassion and to continually improve the standard of care provided to the community through the promotion of value-based quality education and research.

This is the first study to evaluate the financial needs of recipients of financial aid from a multispecialty tertiary care hospital and to assess the impact on QoL and socioeconomic status. Considering that QoL predicts treatment outcomes and hence has predictive value, it is also important for medical decision-making. For instance, it has been discovered that QoL is a significant predictor of survival. This predictive capability demonstrates the need for regular QoL measurement [7]. Economic stability is crucial for retirement health and adaption, and social connectedness is a crucial health-protecting feature. Financial security, social connections, and health are correlated cross-sectionally over time. The results of the current investigation also confirmed all of these traits [8]. A study found that Americans struggling with medical bills had more days with psychological symptoms compared to those without difficulties. However, there were no significant differences in mental health manifestations between 2013 and 2016. Over four years, healthcare coverage improved, but psychiatric disorders increased [9]. Another study examines the link between economic stress and depressive disorders in high-, low-, and middle-income countries. Results show consistent markers of depression, but actual earnings and wealth levels are ambiguous. The study emphasizes collaboration between psychologists and financial professionals in creating targeted interventions to reduce distress or financial anxiety [10]. These findings are congruent with the outcomes of our investigation. According to numerous studies, rehospitalization and death in individuals with illnesses are inversely correlated with QoL. Quality of life is increasingly being utilized in a broader context to evaluate the effectiveness of health services and the results of interventions [11,12]. While the specific details of other studies may vary, there are likely common themes and similarities in research exploring the effect of financial aid on the quality of life for individuals in healthcare settings. Quality of life is an important aspect of medical and health research, and QoL research includes a variety of patient groups and many research methods. Most QOL studies in health and medicine have conceptual and methodological limitations. In general, theories and theoretical frameworks improve the understanding of QoL. One of the findings of a previous study is that people with the maximum well-being or quality of life in the most primary dimensions mostly claim to be happy [13]. Additionally, other studies may similarly investigate the long-term sustainability and effectiveness of financial aid interventions, aiming to understand if positive outcomes persist over time. In a previous study, there was an association between food insecurity, financial well-being, and quality of life [14]. Previous studies aligned that both subjectively and objectively measured financial stress are inversely associated with good self-reported health and quality of life and positively associated with self-reported depression among the elderly [15]. The methodology used in these studies, such as survey tools or interviews to measure quality of life, might also align with the approach taken in this study. Furthermore, similarities may exist in the exploration of policy implications and the potential scalability of successful interventions to inform broader healthcare practices. By identifying and comparing these commonalities, a more comprehensive understanding of the effect of financial aid on the quality of life across various healthcare contexts can be achieved.

Limitations

The study acknowledges potential limitations, such as selection bias due to the voluntary nature of participation. The results may be influenced by external factors, including the broader economic environment and social support systems. The external validity of the study might be undermined due to its limited sample size and the diverse nature of the sampled population, and the study was conducted in a charitable hospital where there is an expectation for financial support. Furthermore, confining the study to Kerala and restricting the selection to only five departments could restrict its applicability to broader contexts.

Recommendations

Psychologists define enhancing QoL as achieving satisfaction across multiple domains, encompassing mental and physical health, social connections and interactions, personal and professional development, and the acquisition of new skills. Upon analyzing the QoL of beneficiaries, it becomes evident that their QoL did indeed improve after receiving financial assistance from the Patient Services Department of AIMS. Therefore, it is recommended to adopt a policy that integrates medical care with the holistic development of individuals, thereby addressing their multifaceted needs comprehensively. Raise people's understanding of the value of QoL and how it connects to general well-being. This can entail holding community gatherings, workshops, or seminars to talk about QoL domains, QoL-influencing variables, and QoL-enhancing techniques. It is important to improve the integration between public and private organizations.

## Conclusions

The findings of this study concluded that there is a significant difference in the QoL of beneficiaries after receiving financial aid from a multi-specialty tertiary care hospital. This study emphasizes the indispensable role of financial aid in healthcare, not only as a means of addressing economic barriers but as a significant determinant of patient well-being. It reaffirms the importance of providing comprehensive, patient-centred care that acknowledges the interdependence of medical treatment and financial stability. By doing so, multi-specialty tertiary care hospitals can better fulfil their mission of ensuring equitable access to healthcare and ultimately contribute to an improved quality of life for all their beneficiaries. These findings have broader implications for healthcare policy and practice, encouraging a more holistic approach to patient support and care.

In 25 years, the hospital has treated 1,96,29,527 patients with amazing success. As of January 2023, AIMS had treated 59,28,728 patients for free or at a reduced cost; this amounted to charitable medical care expenditures of ₹816 crores. People with lower to intermediate incomes receive charity, and the investment yields a many-fold return in the form of improvement in the QoL and, thereby, increased productivity. Financial aid benefits the entire family, even though only one member receives it. Therefore, when comparing the investment in charity, society and the community will profit more in this way. This strategy can be regarded as a ground-breaking step on our part and would surely increase the productivity of public health. From these data, it is proven that the Patient Services Department in AIMS has made a significant impact on the socio-economically backward patients in Kerala.
